# Obstacle praevia atypique: à propos d'un cas

**DOI:** 10.11604/pamj.2014.19.141.5447

**Published:** 2014-10-13

**Authors:** Moncef Chagou, Khadija Bernoussi

**Affiliations:** 1 Service de Gynécologie Obstétrique Cancérologie et Grossesse à haut risque, Maternité Souissi, Université Mohammed V, Rabat, Maroc

**Keywords:** Obstacle praevia, kyste ovarien, césarienne, annexectomie, praevia obstacle, ovarian cyst, c-section, adnexectomy

## Image en medicine

Nous rapportons le cas d'une femme âgée de 25 ans tabagique sous contraceptif oral pendant 2 ans ayant arrêté 6 mois avant la conception. La patiente est une primigeste primipare référée d'une maison d'accouchement pour douleur pelvienne sur une grossesse à terme. A l'admission, la patiente est algique EVA à 7 avec un utérus est relâché à la palpation. Au toucher vaginal, le col est mi-long admettant un doigt, la présentation est céphalique mobile. La poche des eaux est intacte avec une sensation d'une masse bombante au niveau du douglas douloureuse à la mobilisation. A ce stade nous pensions essentiellement à trois diagnostics, un kyste organique, une tumeur des trompes et à un cancer de l'ovaire. Nous avons réalisé une échographie qui a objectivé un énorme kyste dont les mensurations étaient difficiles à mesurer. Une césarienne a été indiquée pour obstacle praevia, l'exploration chirurgicale a trouvé un énorme kyste ovarien tordu de 14 cm/7 cm (2 tours de spires) ( A et B). Nous avons réalisé une annexectomie après extraction d'un nouveau né de sexe féminin poids de naissance 3500g. Les suites opératoires normales.

**Figure 1:**
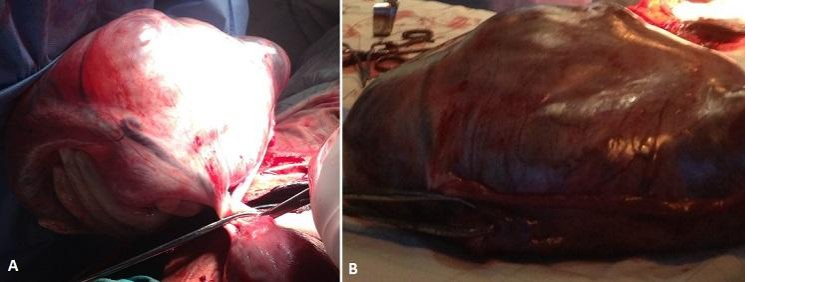
(A) énorme kyste de 14 cm de contenu liquidien non rompu, aspect en faveur. (B) le kyste ovarien après annexectomie d'un kyste séreux

